# Large Device-Related Thrombus Detected following Symptoms of Transient Ischemic Attack

**DOI:** 10.1155/2021/9195984

**Published:** 2021-11-23

**Authors:** Osayi Lawani, Edward Baptista

**Affiliations:** ^1^University of Houston College of Medicine/HCA Houston Healthcare, USA; ^2^Vital Heart & Vein, USA

## Abstract

As an independent risk factor for stroke, atrial fibrillation has been shown to be associated with a fivefold increase in the cause of embolic stroke in comparison to healthy individuals without atrial fibrillation. This risk may be compounded by other factors; however, the main probable cause of stroke leading from atrial fibrillation is thrombus formation in the left atrial appendage. In patients for whom anticoagulation is contraindicated, left atrial appendage occlusion has become a leading alternative option for therapeutic prevention of thromboembolism and stroke in patients with this condition. Unfortunately, these devices (particularly the WATCHMAN) have been associated with a 3-6% incidence of intracardiac thrombus development postimplantation. Some risk factors for the development of device-related thrombus are high platelet count, permanent atrial fibrillation, resistance to clopidogrel, and prior transient ischemic attack or stroke. Despite following an anticoagulant regimen, thrombus formation was reported in 5.6% of participants of a randomized clinical trial, and further analysis showed that some of these patients continued to develop either ischemic stroke or thromboembolism five years later as compared to patients without initial thrombus development. We present a case of an elderly male with prior history of stroke and transient ischemic attack who developed a large device-related thrombus five months following WATCHMAN FLX™ implantation. Currently, there are no specific recommendations on the management of this rare complication; however, we discuss possible consideration of initially prolonging anticoagulation therapy following implantation for high-risk individuals, as there is an increased possibility for thrombus formation in this population. Management options should continue to be studied for therapeutic benefit in streamlining postprocedural therapy and improve future outcomes in the use of left atrial appendage occlusion devices, as well as continual thrombus prevention.

## 1. Introduction

The incidence and prevalence of atrial fibrillation (AF) have increased greatly in the past 20 years as the population of elderly individuals continues to grow, with 75 years being the median age of patients with AF [1]. Currently, there are greater than two million Americans and eight million Europeans that are affected with this condition and is likely underestimated due to lack of notable symptoms [2]. Since the first confirmation of a relationship between an electrocardiogram with AF and a clinical irregularly irregular pulse was published by a group of European physicians in 1909, there has been a continual attempt to find various methods to eliminate or manage this disorder [3]. Management in nonrheumatic AF patients with a CHA_2_DS_2_ − VASc score ≥ 2 consists of an antiarrhythmic agent along with common oral anticoagulants such as vitamin K antagonists, or direct oral anticoagulants via thrombin inhibition, or factor *X*_*a*_ inhibition as to prevent thrombosis [2, 4]. Additionally, in patients with a history of coronary artery disease and planned percutaneous coronary intervention, dual antiplatelet therapy with aspirin and a P2Y_12_ receptor antagonist is included to prevent risk of thrombotic ischemic events and stent thrombosis [2].

In patients for whom anticoagulation is contraindicated, device implantation to close off the left atrial appendage (LAA) is the next likely choice in intervention and has become a leading alternative option for therapeutic prevention of thromboembolism; several devices have been developed for this use (Table 1) [5]. Unfortunately, some of these devices have been associated with about a 3-6% incidence of intracardiac thrombus development postimplantation despite the use of anticoagulant therapy [6]. In this report, we present a case of an elderly male who was found to develop a large device-related thrombus in his left atrium five months following placement of a WATCHMAN device.

## 2. Case History

A 75-year-old male with a past medical history of paroxysmal atrial fibrillation, recent WATCHMAN FLX™ placement, coronary artery disease with three cardiac stents, hypertension, hyperlipidemia, bilateral carotid endarterectomies, recurrent transient ischemic attack (TIA), and two prior cerebrovascular accidents presented to our emergency department with complaints of left-sided numbness that began two hours prior to arrival.

Roughly eight months earlier, the patient experienced similar symptoms and was diagnosed with TIA. He had been taking apixaban 5 mg daily and clopidogrel 75 mg daily following previous cardiac stenting and asserted that he remained compliant with his prescriptions. A transthoracic echocardiogram (TTE) performed showed an ejection fraction of 55-60% and a mildly dilated left atrium. No defects or shunts were noted, and the foramen ovale was not patent. The patient was discharged home and advised to continue his prescriptions for apixaban and clopidogrel.

One month later, the patient returned to an emergency department with complaints of acute onset right-sided weakness and numbness. A magnetic resonance imaging (MRI) of the brain showed ischemic events in multiple territories, and a computed tomography (CT) angiogram of the neck vessels indicated a focal left (dominant) vertebral artery stenosis. Neurointerventional radiology was consulted, and a stenting of the left vertebral artery was performed. Telemetry showed atrial fibrillation with controlled ventricular response. The patient stated that he had remained compliant with taking apixaban and clopidogrel. A platelet P2Y_12_ assay was performed to assess failure of therapy with clopidogrel and was negative. As there was no clear etiology to the diagnosed embolic stroke, a transesophageal echocardiogram (TEE) was ordered. The TEE was deferred to a later date due to patient preference. On discharge, apixaban was changed to rivaroxaban 20 mg daily, and the patient was instructed to continue taking clopidogrel and to follow-up out-patient with his primary cardiologist.

Exactly one month later, the patient was found to be in persistent AF during a follow-up appointment with his cardiologist. The patient also admittedly had several mechanical falls in the past few months. It was decided by his outpatient cardiologist that the patient would undergo implantation of a WATCHMAN FLX™ device for left atrial appendage closure under TEE guidance. The appendage, which was 22 mm in diameter, showed no clot within its interior prior to device implantation. A 27 mm device was ultimately placed more distally following a partial recapture after an initial attempt placed the device somewhat proximal to the opening. A tug test was satisfactory, and no leaks were noted. The patient was instructed to begin taking aspirin 81 mg daily, to continue taking clopidogrel and rivaroxaban, and to return in six weeks for a repeat TEE.

About two months later, the patient had a repeat TEE which showed a normal sized left atrium and a well-positioned WATCHMAN FLX™ without significant peri-device leak (<4 mm). No spontaneous echo contrast, new shunt, or evidence of thrombus was observed. As per protocol, the patient was instructed by his cardiologist to continue taking clopidogrel and aspirin daily, to discontinue use of rivaroxaban, and to follow-up within six months.

**Figure 1:**
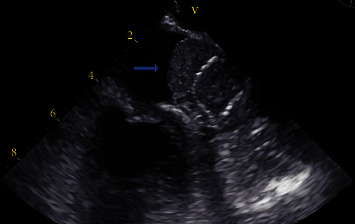
Transesophageal echocardiogram indicating a large thrombus located at the opening of the left atrial appendage covering an implanted WATCHMAN FLX™ device.

**Figure 2:**
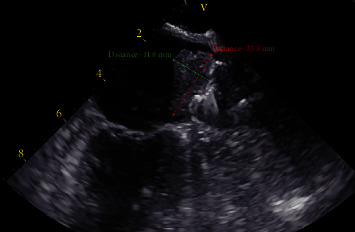
Transesophageal echocardiogram measuring a large thrombus within the left atrium atop an implanted WATCHMAN FLX™ device.

Over three months later, the patient presented to the emergency department at our hospital with complaints of left-sided numbness. It was the patient's first encounter at this facility as he was visiting family from out-of-town. Laboratory results at the time of admission were as follows: creatinine 1.2 mg/dL, calcium 8.6 mg/dL, rapid troponin 0.01 ng/ml, low-density lipoprotein 76.45 mg/dL, high-density lipoprotein 26 mg/dL, hemoglobin 14.7 g/dL, and a platelet count of 124 × 10^3^/uL. Chest X-ray, head CT, brain MRI, and CT angiogram of the head and neck were unremarkable. The patient stated that he had been compliant with taking his prescribed clopidogrel and aspirin, as well as amlodipine 5 mg daily and carvedilol 6.25 mg twice a day. Causes of the patients' symptoms that may have mimicked a TIA (e.g., anxiety, seizure, migraine, and syncope) were unfounded following laboratory results and a bedside evaluation by neurology. As the etiology of the diagnosed TIA was unclear, cardiology was consulted, and a TEE was scheduled to reevaluate the positioning of his previously implanted WATCHMAN FLX™ device for leakage and to rule out any thrombus formation. The TEE showed an ejection fraction of 55-59%, a mildly dilated left atrium, and a well-positioned WATCHMAN FLX™ without peri-device leakage. Surprisingly, it was revealed that there was a large thrombus measuring 2.5 × 1.5 cm at the mouth of the LAA that was adherent to and covered the entire top of the WATCHMAN FLX™ device (Figures 1 and 2). No other structural cardiac defects were noted. The patient was promptly restarted on rivaroxaban and on discharge was instructed to follow-up out-patient with his cardiologist for continued management.

**Figure 3:**
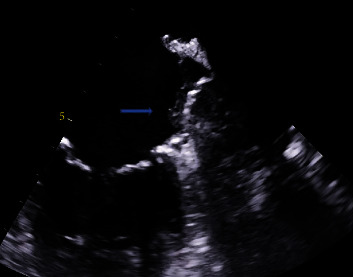
Repeat transesophageal echocardiogram showing a decrease in clot formation on a WATCHMAN FLX™ device within the left atrial appendage.

A little over two months later, the patient returned to his cardiologist for an outpatient follow-up. He denied any further complaints of stroke-like symptoms and continued to take rivaroxaban as prescribed. Another TEE was performed and showed a moderate to severely dilated left atrium. The WATCHMAN FLX™ device was noted in the LAA, as was a small mobile thrombus. The right atrium was also moderately dilated without evidence of thrombus. In comparison to the previous TEE performed over two months prior, the attached thrombus to the device was significantly smaller, but not completely dissolved (Figure 3). The patient was advised to continue rivaroxaban 20 mg daily and to follow-up with his cardiologist for continued surveillance.

## 3. Discussion

As the left atrium dilates from continued fibrillation, there is more stasis and thrombus formation within the LAA [7]. Since the 1990s, interest in treating the LAA in AF patients increased with the advancement of the maze procedure, common use of TEE, and the development of percutaneous occlusion devices [8]. Left atrial appendage occlusion (LAAO) has been the primary method of stroke prevention in AF patients who have a contraindication to anticoagulation therapy, which includes increased risk for fall, lack of patient compliance, discontinuation of anticoagulants due to gastrointestinal bleed or recent stroke, or difficulty in maintaining a therapeutic window (as seen with warfarin use) [7, 8]. Based on both the PROTECT-AF and PREVAIL studies, the WATCHMAN has continually been a popular choice in AF treatment, as it was shown that using this device was not inferior to being on oral anticoagulation to prevent thromboembolism in AF [8].

Changes in the second version of the WATCHMAN FLX in comparison to its predecessor were evaluated during the PINNACLE FLX study and indicated that there was indeed a decreased incidence of complications and increased evidence of effective LAA closure at one year [9]. Device-related thrombosis (DRT) was only found in 3.7% of patients after 12 months of follow-up; however, it was attributed to both the change in the structure of the device and possibly the change in the 6-week postimplantation anticoagulation course regimen from warfarin and aspirin used in the PROTECT-AF and PREVAIL trials, to use of direct oral anticoagulants and aspirin [9]. It was also noted that the new antithrombotic regimen showed an absence of DRT in patients who were compliant with taking these medications [9]. This was not the case for the individual in this report, although his DRT could have had a multifactorial cause.

It is important to mention that although the patient in this report was discovered to have a DRT following a TIA, thrombus is not always associated with an embolic event and is usually an incidental finding. Risk factors for the development of DRT are high platelet count, resistance to clopidogrel, an elevated CHADS_2_ and CHA_2_DS_2_-VASc score, decreased left ventricular ejection fraction, permanent AF, prior TIA or stroke, or device-specific issues (large mean device size or deep implantation) [10]. With regards to this case, the patient met at least three risk factors for thromboembolism formation. The pathophysiology of DRT can be directly related to elderly individuals with prior history of stroke, elevated CHA_2_DS_s_-VASc score, and vascular disease, all of which are indicated in an increased prothrombotic state [11]. DRT may also be induced by incomplete or deep implantation of the device by exposing areas of trabeculations [11]. Large devices also increase the risk for DRT due to having an increased fabric surface area [11]. The patient in this case had his device implanted deeply on a second attempt and also had a device placed that was 5 mm larger than the diameter of his LAA.

The most common cause of DRT has been noted to occur following the discontinuation of oral anticoagulant therapy [6]. At this time, there is no indication of which oral anticoagulant is preferred as one retrospective study suggested that there was no significant difference in developing DRT with the initial use of either vitamin K antagonists or direct oral anticoagulants [11]. What is known is that a recent analysis showed that after five years, about 25% of patients who were diagnosed with DRT from the PROTECT-AF and PREVAIL studies later developed either ischemic stroke or thromboembolism compared to patients without DRT, and that there was also a temporal association [11]. Although the incidence of DRT is about 3-7%, it is associated with a threefold increase in stroke risk [11]. Furthermore, as there is usually resolution of DRT with the use of direct oral anticoagulation, there have been several accounts of recurrent DRT after anticoagulation was discontinued [12]. Consequently, more thoughtful consideration should be placed for patients that qualify for an LAAO device and have a significant history of stroke or thromboembolism.

**Figure 4:**
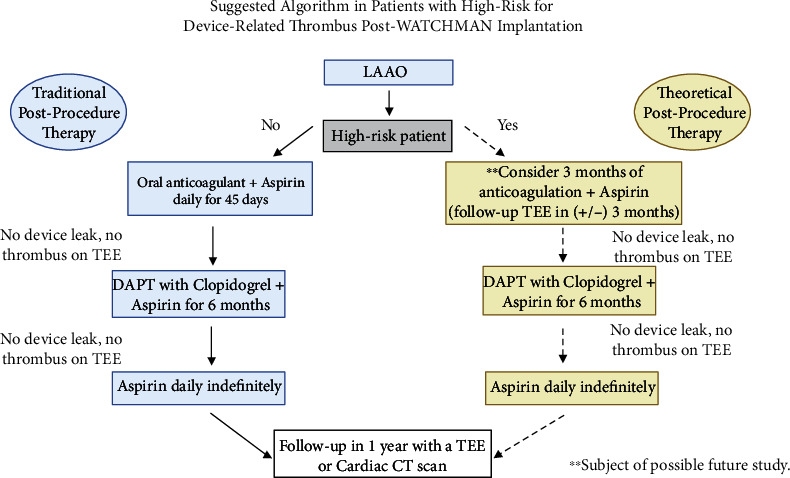
Suggested algorithm for assessment and treatment of a patient post-WATCHMAN placement who may be high risk for DRT. LAAO: left atrial appendage occlusion; TEE: transesophageal echocardiogram; DAPT: dual antiplatelet therapy; DRT: device-related thrombus; CT: computed tomography [11].

For high-risk patients prior to LAAO device placement, it is proposed that anticoagulation continue for >45 days following implantation, then a repeat TEE is performed at a later date, and the patient can start dual antiplatelet therapy followed by aspirin indefinitely if there is no evidence of peri-device leaks or DRT [13]. Future studies should focus on an optional length of time a high-risk patient should stay on anticoagulation postimplantation as this is presently undetermined (Figure 4) [11].

Duration of use and choice of anticoagulant and antiplatelet regimen should be tailored for high-risk patients. In addition, it may be worth investigating whether P2Y_12_ inhibitors other than clopidogrel should be used for patients who are prone to thromboembolism as they have better platelet aggregation inhibition during the device sealing process [13]. Previously, a DRT score based on predictors was suggested to assist in identifying individuals that would be at a high risk for thrombus development postdevice implantation. This would be helpful as it would potentially lead to finding whether factors related to anticoagulation, the patient, or the actual procedure contributed to the formation of DRT [12]. At this time, treatment strategies for high-risk individuals who may develop DRT have not been clinically proven and should be treated on a case-by-case basis.

## 4. Conclusion

Although the incidence of DRT following WATCHMAN implantation is relatively low, it does have a significant impact on how high-risk patients are viewed and considered for treatment. As the results of the ASAP-TOO trial will indicate whether anticoagulation is needed overall after device implantation, the incidence of DRT should continue to be further investigated, particularly for high-risk individuals. Prolonging anticoagulation or change in antiplatelet regimen should also continue to be studied for therapeutic benefit. One study suggested possible coating of the device in an antithrombotic agent to help in decreasing the risk of DRT development [12]. Integration of these changes into clinical practice will likely assist in streamlining postprocedural therapy as to keep up with achieving continual promising outcomes with the use of LAAO devices, as well as continual thrombus prevention.

## Figures and Tables

**Table 1:** Devices for use in percutaneous left atrial appendage occlusion [5].

Device	Manufacturer	Design	Sizes (mm)	Sheath (F)	Approval status
*Endocardial LAAO devices*					
WATCHMAN	Boston Scientific	Single (lobe)	21, 24, 27, 30, 33	14	CE Mark (2005) FDA (2015)
WATCHMAN FLX			20, 24, 27, 31, 35	14	CE Mark (2019) FDA (2020)
Amplatzer Cardiac Plug	Abbot Vascular, formerly St. Jude Medical	Double (lobe and disc)	16, 18, 20, 22, 24, 26, 28, 30	9-13	CE Mark (2008)
Amplatzer Amulet			16, 18, 20, 22, 25, 28, 31, 34	12-14	CE Mark (2013)
WaveCrest	Biosense Webster, Inc. (Johnson and Johnson company)	Single (lobe)	22, 27, 32	12	CE Mark (2013)
Occlutech	Occlutech International AB	Single (lobe)	15, 18, 21, 24, 27, 30, 33, 36, 39	12, 14	CE Mark (2016)
LAmbre	Lifetech Scientific Co., Ltd.	Double (umbrella and cover)	16, 18, 20, 22, 24, 26, 28, 30, 32, 34, 36	8-10	CE Mark (2016); CFDA (2017)
Sideris transcatheter patch	Custom Medical Devices	Frameless, bioabsorbable, balloon-deliverable device	15-25	13	Undergoing clinical evaluation
Ultraseal	Cardia, Inc.	Double (bulb and sail)	16, 18, 20, 22, 24, 26, 28, 30, 32	10-12	CE Mark (2016)
SeaLA	Hangzhou Valued Medtech Co., Ltd.	Double (dual disc)	16, 18, 20, 22, 24, 26, 28, 30, 32, 34, 36	9-12	Undergoing clinical evaluation
LeFort	Lepu Medical Technology (Beijing) Co., Ltd.	Single (lobe)	21-33	—	Undergoing clinical evaluation
Pfm device	Pfm Medical	Single (disc), dual anchor	—	10-12	Undergoing clinical evaluation

*Epicardial LAAO devices*					
Lariat	SentreHeart, Inc.	Endoepidcardial	40 (W), (45 [W] lariat +) x 20 (H) x 70 (L)	12	CE Mark (2015); FDA 510(k) (2006), surgical use only
Sierra	Aegis Medical Innovations Inc.	Epicardial	Single size	20	Undergoing clinical evaluation

LAAO: left atrial appendage occlusion; CE Mark: Comformité Europëenne Mark; CFDA: China Food and Drug Administration; FDA: United States Food and Drug Administration; W: width; H: height; L: length.

## Data Availability

Data is available upon request to the corresponding author.

## References

[1] Chugh S. S., Blackshear J. L., Shen W.-K., Hammill S. C., Gersh B. J. (2001). Epidemiology and natural history of atrial fibrillation: clinical implications. *Journal of the American College of Cardiology*.

[2] Capodanno D., Huber H., Mehran R. (2019). Management of Antithrombotic Therapy in Atrial Fibrillation Patients Undergoing PCI:. *Journal of the American College of Cardiology*.

[3] Fazekas T. (2007). The concise history of atrial fibrillation. *Orvostörténeti Közlemények*.

[4] de Backer O., Arnous S., Ihlemann N. (2014). Percutaneous left atrial appendage occlusion for stroke prevention in atrial fibrillation: an update. *Open Heart*.

[5] Asmarats L., Rodes-Cabau J. (2018). The spectrum of devices for percutaneous left atrial appendage occlusion. *Cardiac Interventions Today*.

[6] Hannawi B., Beg F., Valderrabano M., Kurrelmeyer K. (2021). Device-related thrombus: a reason for concern?. *Methodist Debakey Care*.

[7] Romero J., Perez I. E., Krumerman A., Garcia M. J., Lucariello R. J. (2014). Left atrial appendage closure devices. *Clinical Medicine Insights: Cardiology*.

[8] Chatterjee S., Alexander J. C., Pearson P. J., Feldman T. (2011). Left atrial appendage occlusion: lessons learned from surgical and transcatheter experiences. *The Annals of Thoracic Surgery*.

[9] Kar S., Doshi S. K., Sadhu A. (2021). Primary outcome evaluation of a next-generation left atrial appendage closure device. *Circulation*.

[10] Dukkipati S. R., Kar S., Holmes D. R. (2018). Device-related thrombus after left atrial appendage closure. *Circulation*.

[11] Turagam M., Reddy V. Y., Dukkipati S. (2018). Device-related thrombus: understanding and managing the “Achilles heel” of LAA closure. *American College of Cardiology*.

[12] Alkhouli M., Holmes D. R. (2020). Remaining challenges with transcatheter left atrial appendage closure. *Mayo Clinic Proceedings*.

[13] Garot P., Cormier B., Horvilleur J. (2019). Device-related thrombus after left atrial appendage closure. *International Cardiology Review*.

